# The Clinical Significance of Iron Overload and Iron Metabolism in Myelodysplastic Syndrome and Acute Myeloid Leukemia

**DOI:** 10.3389/fimmu.2020.627662

**Published:** 2021-02-19

**Authors:** Sarah Weber, Anastasia Parmon, Nina Kurrle, Frank Schnütgen, Hubert Serve

**Affiliations:** ^1^ Department of Medicine, Hematology/Oncology, University Hospital Frankfurt, Goethe University, Frankfurt am Main, Germany; ^2^ German Cancer Consortium (DKTK), Partner Site Frankfurt/Mainz and German Cancer Research Center (DKFZ), Heidelberg, Germany; ^3^ Frankfurt Cancer Institute, Goethe University, Frankfurt am Main, Germany

**Keywords:** myelodysplastic syndrome, acute myeloid leukemia, iron overload, reactive oxygen species, microenvironment, iron chelation

## Abstract

Myelodysplastic****syndrome (MDS) and acute myeloid leukemia (AML) are clonal hematopoietic stem cell diseases leading to an insufficient formation of functional blood cells. Disease-immanent factors as insufficient erythropoiesis and treatment-related factors as recurrent treatment with red blood cell transfusions frequently lead to systemic iron overload in MDS and AML patients. In addition, alterations of function and expression of proteins associated with iron metabolism are increasingly recognized to be pathogenetic factors and potential vulnerabilities of these diseases. Iron is known to be involved in multiple intracellular and extracellular processes. It is essential for cell metabolism as well as for cell proliferation and closely linked to the formation of reactive oxygen species. Therefore, iron can influence the course of clonal myeloid disorders, the leukemic environment and the occurrence as well as the defense of infections. Imbalances of iron homeostasis may induce cell death of normal but also of malignant cells. New potential treatment strategies utilizing the importance of the iron homeostasis include iron chelation, modulation of proteins involved in iron metabolism, induction of leukemic cell death *via* ferroptosis and exploitation of iron proteins for the delivery of antileukemic drugs. Here, we provide an overview of some of the latest findings about the function, the prognostic impact and potential treatment strategies of iron in patients with MDS and AML.

## Introduction

Myelodysplastic syndrome (MDS) and acute myeloid leukemia (AML) represent heterogeneous clonal hematopoietic stem cells disorders. MDS is characterized by dysplasia of hematopoietic cells, AML by uncontrolled proliferation of poorly differentiated hematopoietic cells (blasts). Both diseases lead to insufficient hematopoiesis. Chronic fatigue due to anemia, bleeding due to thrombocytopenia and infection due to neutropenia are typical consequences. MDS bone marrow is prone to leukemic transformation with approximately 30% of MDS patients developing secondary AML over time (1). AML, being the most common acute leukemia in adults, is a disease that in most cases needs immediate treatment to avoid death within months or even weeks. Although our knowledge about the molecular drivers of AML is rapidly increasing, and recently resulted in the development of novel drugs and of molecularly informed treatment stratification, the 5-year overall survival (OS) rate is still below 30% (2).

**Figure 1:**
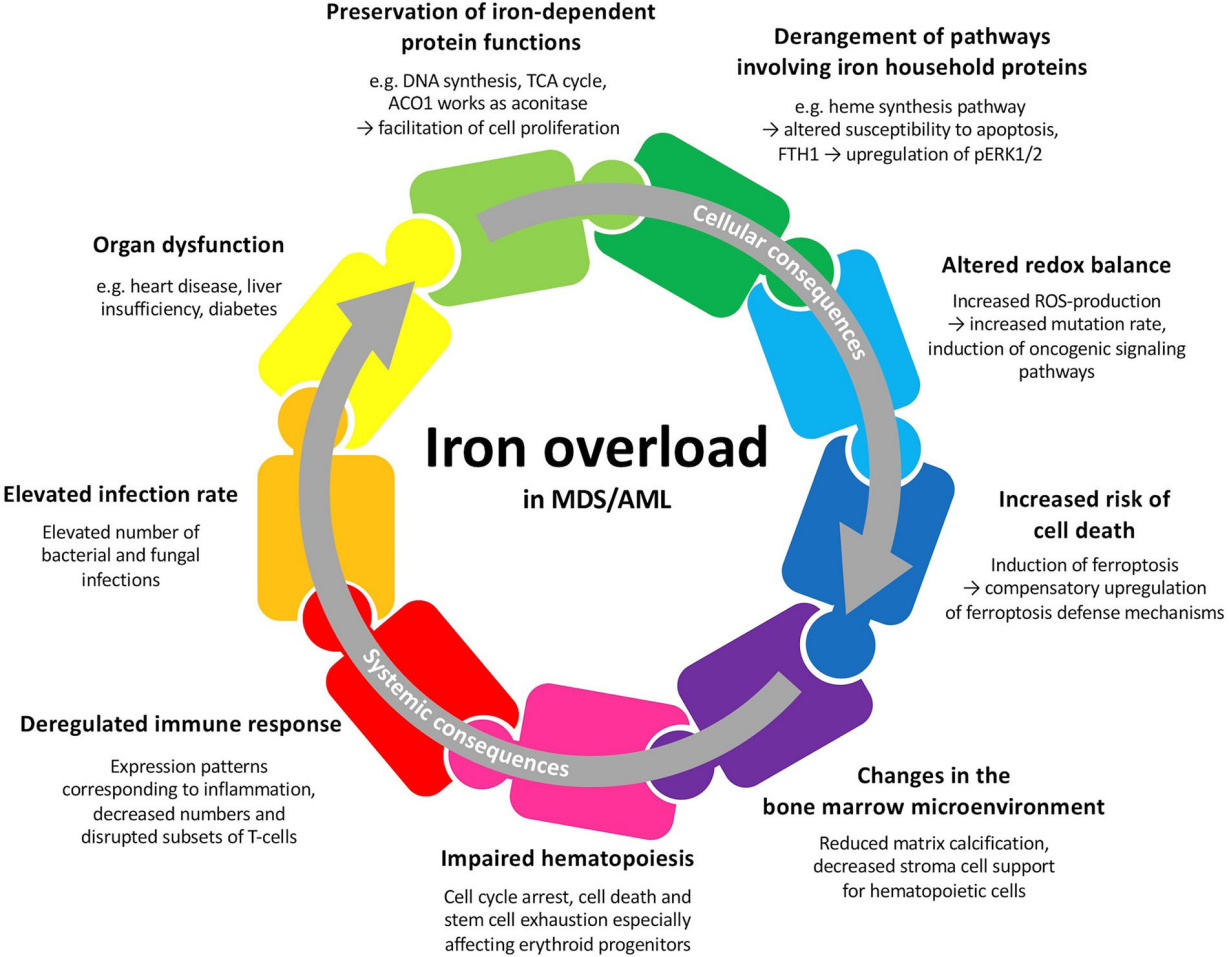
Potential cellular and systemic consequences of iron overload in patients with MDS or AML. Many of these factors are interwoven and may all together contribute to patient outcome.

MDS and AML patients may develop primary iron overload arising from insufficient erythropoiesis (3). Repeated transfusions, which aim at ameliorating the symptoms of anemia, often lead to secondary iron overload. Iron overload in MDS and AML patients may lead to multiple cellular and systemic changes and therefore plays a crucial role in these hematologic malignancies (**Figure 1**). Besides the importance of iron and proteins involved in iron metabolism for multiple cellular functions, iron is tightly connected to the production of reactive oxygen species (ROS) and can lead to cell death when in excess (4). Iron overload in the bone marrow and other tissues can result in alterations of the microenvironment and contribute to increased morbidity (5). In this respect, iron has been demonstrated to participate in aggravating the symptoms of MDS and AML patients by contributing to bone marrow failure (6). Excess iron can also alter the components of the immune system and result in an increased susceptibility to various infections (7). Therefore, serum and cellular iron levels have a prognostic value at initial diagnosis, might influence the response to chemotherapy and predict the outcome after hematopoietic stem cell transplantation (HSCT) (8–10). The involvement of iron in diverse metabolic processes and its special necessity for malignant cells makes it an interesting therapeutic target (11).

In this review, we will first give an overview of the molecular basis of iron metabolism and its role in hematopoiesis. We will then focus on the altered iron metabolism in MDS and AML patients including clinical consequences. Subsequently, we will elucidate the effect of iron overload on the pathophysiology of MDS and AML, clinical consequences of the altered iron metabolism and its use as a potential target for therapy.

## Iron Homeostasis and its Role for Normal Hematopoiesis

Iron is an essential micronutrient for fundamental metabolic processes in all cells and organisms and is therefore a crucial element for terrestrial life. A vital iron-binding protein of the human body is hemoglobin, which is crucial for the transport, storage and distribution of oxygen. Hemoglobin in circulating erythrocytes and erythroid precursors in the bone marrow contains about two thirds of the total body iron (12). Besides, iron is bound to myoglobin in the muscles. Iron is also part of prosthetic groups such as in cytochrome proteins and Fe-S clusters due to its ability to facilitate electron transfer. Thereby, it is essential for the function of the citric acid cycle (TCA), the respiratory chain, DNA synthesis and DNA repair.

**Figure 2:**
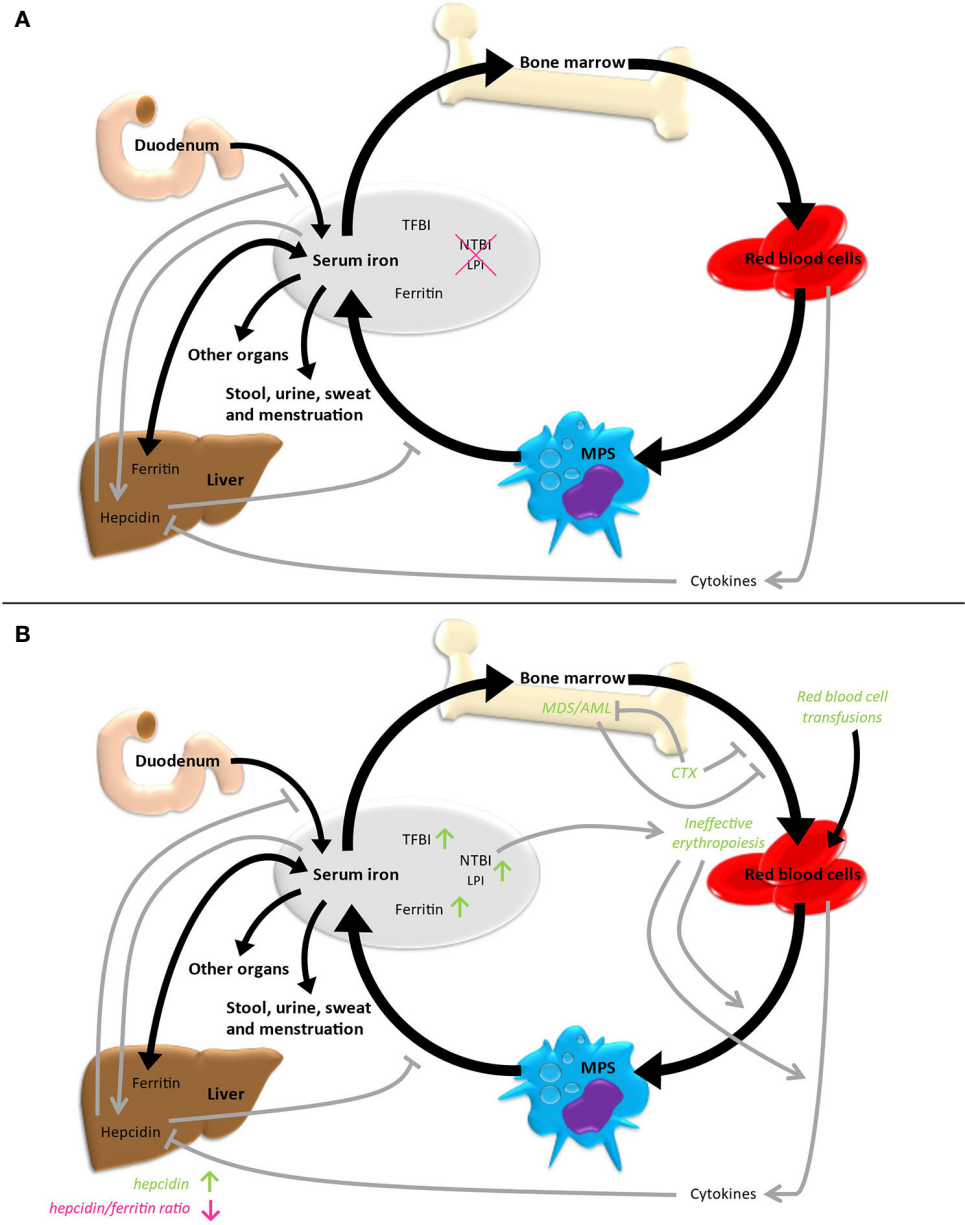
Iron metabolism under physiological conditions **(A)** and in case of MDS/AML **(B)**. Black arrows indicate direct iron metabolism, gray arrows represent regulatory mechanisms. LPI, labile plasma iron; MPS, mononuclear phagocyte system; NTBI, non-transferrin-bound iron; TFBI, transferrin-bound iron.

Systemic iron homeostasis is maintained by a balance of iron uptake, recycling and loss (**Figure 2A**). Nutritional iron is mainly available as ferric iron, which can be reduced by ferrireductases. Subsequently, ferrous iron can be internalized into enterocytes *via* need-oriented gastrointestinal active transport mechanisms by the divalent metal ion transporter (SLC11A2). Iron may also be internalized through siderophore-associated binding to lipocalin-2 (LCN2) and subsequent endocytosis (13). Moreover, nutritional heme and possibly also ferritin can be absorbed by enterocytes *via* mechanisms not fully determined yet (14). Efflux of iron across the basolateral membrane into the bloodstream *via* ferroportin (SLC40A1), the only known iron exporter, is usually followed by its oxidation to ferric iron by the membrane-bound ferroxidase hephaestin. Ferric iron can be loaded to transferrin (TF) and then be used for the needs of the body. Excess iron is stored *via* ferritin (FTH and FTL) mainly in the liver. The body loses iron *via* exfoliation of cells on the inner and outer surfaces of the body with stool, urine, sweat and blood loss in menstruating women, but there are no physiological active excretion mechanisms to release an excess of iron in mammals and humans and the iron excretion cannot physiologically be increased beyond these values. High iron levels lead to systemic secretion of hepcidin, the most relevant regulator of the systemic iron metabolism, by the liver. Hepcidin binds to ferroportin on enterocytes and iron-storing cells like macrophages, resulting in an internalization and degradation of the hepcidin-ferroportin complex and thus effectively shuts down nutritional iron absorption and iron release from internal iron storage. Hepcidin expression is controlled by regulatory feedback mechanisms that involve active erythropoiesis: erythroblast-derived erythroferrone (ERFE), growth differentiation factor 11 (GDF11), growth differentiation factor 15 (GDF15) and twisted gastrulation protein homolog 1 (TWSG1) have been shown to influence hepatic hepcidin secretion, thus linking erythropoietic iron demand to iron supply (15–18).

Overall, only 4% to 10% of the daily iron need is supplied by uptake of nutritional iron, whereas the majority of iron gets recycled by different cell types originating from the bone marrow. Cells within the mononuclear phagocyte system (MPS) remove senescent blood cells *via* phagocytosis and digestion. Afterwards, iron is released into the blood, from where it is transported by transferrin back to the bone marrow for recurrent use in hematopoiesis. About ten times the amount of serum transferrin iron is recycled through this bone marrow-MPS-bone marrow cycle per day (19).

Intracellular iron acquisition is provided by ferrous iron importers (SLC11A2, SLC39A8, SLC39A14) or by binding of diferric transferrin to the cell surface transferrin receptors (TFR: TFRC and TFR2α) resulting in an internalization of the complex by clathrin-mediated endocytosis. Acidification of the endosome results in the release of ferric iron from transferrin. Additionally, circulating FTH can bind transferrin-independently to TFRC and be internalized in this way (20). Endosomal ferric iron is reduced to ferrous iron *via* ferrireductases. Ferrous iron can then be transported to the cytosol, where it represents the labile cellular iron (LCI) pool. This non-bound, redox-active and chelatable iron pool can be utilized in cellular metabolic processes, or, when in excess, be stored in ferritin or excreted *via* ferroportin. NCOA4 can mediate ferritinophagy, while it is degraded *via* HERC2 ubiquitination-mediated induction of the proteasomal degradation machinery in the presence of iron (21). Intracellular iron proteins are post-transcriptionally regulated by the IRP/IREs regulatory network. Therefore, mRNAs of regulated proteins harbor specific hairpin stem loops, called iron-responsive elements (IRE), situated in the 3’ or 5’ untranslated regions. In iron-deplete cells, the iron-responsive element binding proteins ACO1 and IREB2 bind to the IREs of specific mRNAs resulting in mRNA stabilization or translational repression of these mRNAs. In this way, they modulate the expression of iron-regulating proteins, which subsequently leads to an increase of the labile iron pool. In iron-replete cells, ACO1 works instead as aconitase in the TCA cycle and IREB2 undergoes SCF^FBXL5^ E3 ubiquitin ligase mediated ubiquitination and proteasomal degradation (22).

Both, iron deficiency and iron overload lead to impaired hematopoietic functions. Iron deficiency resulting in microcytic anemia due to impaired hemoglobin production is a common nutritional deficiency disorder affecting especially women and children worldwide. As a consequence of iron overload, dysplastic changes and detrimental effects on erythroblast differentiation and maturation resulting in a reduction of the proliferative capacity of erythropoiesis and of erythroblast apoptosis *in vitro* have been described (3, 23). Additionally, iron overload has been shown to induce growth arrest and cell death due to oxidative stress *via* ROS-mediated activation of p38MAPK, JNK and p53 pathways in immature hematopoietic cells (24, 25). Thereby, the IRP/IRE regulatory network is essential in maintaining hematopoietic stem cells in their physiological self-renewal process. While Ireb2(-/-) mice develop microcytic anemia, deletion of Fbxl5 in murine hematopoietic stem cells leads to impaired hematopoiesis due to Ireb2 overexpression and subsequent iron overload (26, 27).

## Elevated Iron Levels in MDS and AML Patients

Measurement of a patient’s iron status is difficult due to various pitfalls of the available methods. Most commonly, iron status is measured based on serum iron indicators such as serum ferritin, transferrin saturation and soluble transferrin receptor (sTFR). However, the results may be influenced by external factors including inflammation, growth factors and organ dysfunctions (28). In case of acute iron overload, exceedance of the transferrin binding capacity leads to detectable amounts of non-transferrin-bound iron (NTBI) in the serum. A subfraction of NTBI is chemically labile plasma iron (LPI), which is toxic due to its redox-activity and can cause oxidative damage to cellular membranes, proteins and DNA (29). NTBI including LPI are loosely bound to serum components as albumin and citrate (30). Thereby, the presence and dynamics of active iron forms as NTBI and LPI may be accountable for direct toxic effects, whereas steady iron markers as ferritin may reflect mainly systemic changes in iron metabolism. Iron overload can also be measured *via* organ biopsies or imaging methods as biomagnetic susceptometry or magnetic resonance imaging (MRI) although these methods are rarely applied due to their invasiveness, costs or insufficient validation (31).

Using this variety of methods, over the years several characteristics of an altered iron metabolism in MDS and AML patients have been found together indicating a state of iron overload in these diseases (**Figure 2B**).

The most common reason for iron overload in patients with hematologic diseases is the administration of multiple red blood cell transfusions representing a massive excess of iron uptake with about 200 mg of iron in one unit of packed red blood cells (32). Transfusion-associated iron is processed by hepatic and splenic macrophages, which recycle heme iron from erythrocytes and release it into the extracellular space, thereby increasing the serum iron pool.

Independent of red blood cell transfusions, disease-immanent factors can contribute to the iron overload phenotype. Dysplastic ineffective erythropoiesis is one of the cardinal manifestations of MDS, leading to an insufficient production of mature erythrocytes and potentially to a higher turnover of erythroid progenitors. This insufficient erythropoiesis leads to the secretion of hepcidin-suppressing cytokines and thus might result in further iron overload. A vicious cycle is formed, in which primary bone marrow dysfunction causes iron overload, which in turn amplifies bone marrow dysfunction. The presence of this mechanism is supported by data from Cui et al., who found elevated hepcidin and ferritin levels, but a reduced hepcidin/ferritin ratio compared to healthy controls in a study including 107 MDS patients without prior transfusions (33). In the same study, elevated ferritin levels correlated with decreased proliferative potential of erythropoiesis *ex vivo*. However, the extent of these mechanisms seems to differ between MDS subtypes. MDS subtypes with a high presence of ring sideroblasts (RARS, corresponding to MDS-RS according to the present WHO classification) as a morphological correlate of iron-loaded mitochondria, have been shown to have the lowest hepcidin/ferritin ratio (34, 35). Therefore, inefficient erythropoiesis might be more prominent in these subtypes than in other MDS patients. Correspondingly, MDS-RS is typically associated with a mutation in the splicing factor gene *SF3B1*. An *SF3B1* mutation was recently identified by Bondu et al. to lead to the expression of an alternative *ERFE* transcript, which suppresses hepcidin transcription and thereby provides an explanation for the increased iron load especially in these patients (36). The European MDS registry (EUMDS) investigated the occurrence of iron overload in MDS patients prospectively (37). Here, clinical data and iron metabolism-associated parameters including serum levels of ferritin, transferrin, hepcidin, GDF15, sTFR, NTBI and LPI were analyzed in newly diagnosed lower-risk MDS patients from 148 centers in 16 countries in Europe and Israel since 2008. The results indicate that the above-mentioned concept of primary, disease-immanent iron overload may not be of strong relevance for the majority of MDS patients: markers of iron overload were elevated over all MDS subtypes. However, occurrence specifically correlated with transfusion-dependent MDS and with the MDS-RS subtype.

During chemotherapy and foremost during hematopoietic stem cell transplantation (HSCT) the iron homeostasis might be further disturbed as a result of erythroid cell lysis and suppressed erythropoiesis. This theory matches data from the German prospective multicenter study ALLIVE including 22 MDS and 90 AML patients and some smaller studies, which show an increase in NTBI and LIP levels during allogeneic HSCT (38–40).

During the course of AML, signs of iron overload have also been described. Frequently, serum ferritin is elevated at initial AML diagnosis. The extent correlates with the leukemic burden, normalizes in remission, and increasing levels may signify a relapse (41). Increased hepcidin serum levels at diagnosis and pre- as well as post-HSCT were described in two small cohorts including exclusively or mostly AML patients (42, 43). However, hepcidin and ferritin are acute-phase proteins and might not only indicate iron overload but may also reflect a state of inflammation. Correspondingly, ferritin and hepcidin serum levels in one of these studies correlated with serum levels of CRP and IL-6 (42). In another study, ferritin levels were also elevated in CRP-low patients and ferritin and hepcidin levels correlated with the number of blood transfusions (43). Overall, valid data including definitive measures of iron overload and investigations in the systemic iron state in AML are missing. Specifically, there are no data available from investigating the interplay of insufficient hematopoiesis and iron metabolism in AML. Presumably, ineffective erythropoiesis due to dysplastic changes applies only to an AML subgroup (especially AML with myelodysplasia-related changes), whereas in other AML subtypes, insufficient erythropoiesis may rather be driven by other pathomechanisms as the suppression of erythropoiesis by inflammatory cytokines (44).

Signs of iron overload show a prognostic impact in both, MDS and AML patients in many studies. One of the open questions in the field is, whether iron overload is just a consequence of increased transfusion frequency, which is a well-known measure of disease severity, which would explain the worse prognosis, or, whether iron overload *per se* has a negative impact on the course of the disease. In both diseases, the degree of transfusion dependency was associated with a worse patient outcome (45–48). However, high levels of LPI were associated with inferior overall and progression-free survival in lower-risk MDS patients irrespective of the transfusion status in the study of the European MDS registry (37). The ALLIVE study revealed that in patients undergoing allogeneic HSCT, pretransplant NTBI was associated with an increased incidence of non-relapse mortality and a worse overall survival, which is hard to explain by the pretransplant disease severity alone (38). Besides, high serum ferritin levels at AML diagnosis were associated with a worse outcome (9, 49, 50). The same is true for ferritin levels before and after allogeneic HSCT in cohorts including mainly MDS and AML patients (51, 52). Data on the prognostic impact of the liver iron content measured by MRI for patients receiving allogeneic HSCT are ambiguous. While a meta-analysis of four studies with mixed patient cohorts including overall 50% AML and 16% MDS patients found that increased liver iron was not indicative for bad patient outcome (53), the ALLIVE study showed an association of high pretransplant liver iron with increased early non-relapse mortality (NRM) (38). Despite different compositions of the patient cohorts with older, more severely iron-overloaded patients in the ALLIVE study, the role for liver iron overload in NRM remains inconclusive.

Taking the data on the prognostic impact of different iron overload markers together, the overall correlation with patient outcome is striking. However, it is difficult to exclude that this is merely the reflection of disease severity. Despite these doubts, clinical correlation data and studies on the consequences of iron overload from other diseases, led to the widespread recommendation to treat transfusion-induced iron overload in patients with hematological malignancies. Several therapeutic options are available that will be reviewed in section *Therapies Aiming at Iron Metabolism as a Possible Target in MDS and AML*.

## Potential Roles of Iron-Related Intracellular Proteins in AML and MDS

To further understand the iron metabolism in MDS and AML, investigating the role of iron-related intracellular proteins might help explaining the interplay between iron and essential intracellular networks in MDS and AML cells.

Expression of iron-importing proteins might be an indicator for the iron need of the cells. It has been appreciated for almost 40 years that AML cells strongly bind to transferrin (54). In humans, two transferrin-binding receptors have been identified: TFRC is a ubiquitously expressed high affinity receptor and TFR2α is restricted to certain cell types as hepatocytes and erythroblasts and has an approximately 25-fold lower affinity for transferrin than TFRC (55, 56). The alternative TFR2 isoform, TFR2β, lacks the transmembrane and cytoplasmic domain but might be involved in the regulation of iron efflux in the MPS (57). Overexpression of TFRC was demonstrated in AML cells (58–60) and supports the hypothesis of a higher iron consumption of these cells. Thereby, TFRC expression was higher in undifferentiated than in more differentiated AML subtypes and decreased with terminal differentiation (59, 61). Neither high *TFRC* mRNA nor TFRC protein levels in AML cells correlated with patient outcome although a correlation was found with increased anemia, thrombopenia and complex cytogenetics (62, 63). On the contrary, higher *TFR2* mRNA levels in bone marrow samples were surprisingly associated with a favorable outcome in AML and MDS patients (64, 65). However, the increase of TFR2 mRNA levels in MDS and AML bone marrow samples were shown to roughly correlate with the proportion of erythroid cells in the marrow and might therefore only to a minor extent reflect the expression of MDS or AML cells themselves (13, 66). This association with the erythroid cell number might be the explanation for the favorable outcome. Deducing from these data, higher TFRC expression of AML cells might reflect an undifferentiated blast status whereas higher TFR2 mRNA expression in the bone marrow of MDS and AML patients might be a marker for the number of erythroid cells. However, there is also evidence for a need of higher iron amounts due to overall higher TFRC expression and the necessity of TFR for leukemic cell growth as shown in TFR antibody studies described in section *Perspectives*.

Only recently, the roles of LCN2 and BDH2 have attracted attention in MDS and AML patients. LCN2 can bind to siderophores and thereby lead to iron internalization *via* endocytosis or to the secretion of iron *via* endosome recycling thereby potentially enabling iron overload or iron deficiency (67). BDH2 catalyzes the rate-limiting step for the formation of the mammalian siderophore 2,5-dihydroxybenzoic acid (68). This might facilitate LCN2-mediated iron uptake but also prevent iron overload in the cytoplasm and iron depletion in mitochondria. In cytogenetically normal AML patients, *LCN2* mRNA was reduced (69). Thereby, high *LCN2* mRNA expression in the bone marrow was associated with a favorable outcome especially in combination with wild-type *FLT3* showing an enhanced apoptosis under hydrogen peroxide and cytarabine treatment whereas showing a protective effect under DFO treatment. On the contrary, *BDH2* overexpression has been associated with poor overall survival in cytogenetically normal AML (70) and with elevated ferritin levels as well as an increased risk for progression to leukemia in MDS (71). As further mechanistical analyses are missing, it can only be speculated that in this case *LCN2* overexpressing cells might have an increased LPI pool predisposing them to oxidative stress, whereas *BDH2* overexpression might prevent cytoplasmatic iron overload. Further studies validating these results and unraveling the underling mechanisms are highly needed.

The intracellular conversion of insoluble ferric to soluble ferrous iron is mediated by ferric reductases including STEAP protein members. Although STEAP1 has no iron reducing function, it co-localizes with transferrin and TFRC suggesting also a role in iron homeostasis. In AML, *STEAP1* was shown to be overexpressed and associated with an adverse OS (72).

Systemically elevated levels of the iron storage protein ferritin suggest a role for intracellular ferritin levels in MDS and AML as well. FTH1 was reported to be expressed particularly in erythroid blasts measured by immunohistochemistry (73). In another study, *FTH1* and *FTL* mRNA overexpression and FTH1 protein overexpression measured by immunoblot were shown in AML primary cells compared to peripheral mononuclear cells (9). The presence of ferritin may reduce the LPI pool and therefore prevent ROS formation. In line with this, a decreased *in vitro* cytotoxic activity of cytarabine was detected in FTH1 overexpressing AML. Additionally, analyses of the erythroleukemia cell line K562 indicate that *FTH1* expression might prevent ROS-induced protein misfolding (74) and ROS-induced activation of the HIF1A/CXCR4 pathway leading to an epithelial-to-mesenchymal transition (EMT)-like phenotype (75). Besides, FTH1 might regulate RAF1 downregulation and activate pERK1/2 through downregulation of the expression of distinct microRNAs (76). Therefore, intracellular ferritin expression might play a role in MDS and AML especially in erythroid blasts on many levels.

Expression of the iron exporter FPT is also suggested to reduce the LPI pool and thereby the formation of ROS. Low *FPT* levels in AML cells correlated with good risk cytogenetics, increased sensitivity to cytarabine treatment and favorable outcomes (10) but a causal relationship could not be deduced from this data.

Overall, several changes in proteins associated with iron metabolism have been detected in MDS and AML cells. Mutually, the iron status and these proteins as well as several intracellular signaling pathways influence each other. Thereby, especially proteins directly regulating the intracellular iron pool seem to have an impact on cell viability and patient outcome.

## Iron and ROS Homeostasis in Leukemogenesis

Iron and ROS homeostasis are closely entangled. Iron contributes to ROS formation by the production of hydroxyl radicals *via* the Haber-Weiss and Fenton reaction. Moreover, iron is involved in indirect ROS production. Multiple iron-containing enzymes and those which require iron as an indispensable cofactor contribute to ROS production under normal conditions (77). So, as an important component of the respiratory chain iron is involved in the formation of mitochondrial ROS during oxidative phosphorylation (78). Vice versa, ROS can interact with iron sulfur clusters ([4Fe-4S]), turning them into their inactive form ([3Fe-4S]+). This leads to a switch in the function of the iron-sulfur cluster protein ACO1 from its role as aconitase in the TCA cycle to its function as an IRE-binding protein regulating the expression of various proteins involved in iron metabolism and other pathways (79).

Elevated ROS levels have been detected in MDS and AML patients compared to controls (80, 81). Moreover, iron overload is accompanied by increased ROS levels in this patient cohort (82–84). Therefore, iron may contribute to leukemogenesis *via* its effect on the ROS homeostasis.

Due to this connection, iron overload has been discussed to be involved in mutagenesis and leukemic transformation. Highly reactive hydroxyl radicals can directly interact with DNA leading to DNA damage (85). Moreover, ROS can stimulate the generation of lipid peroxyl radicals especially of polyunsaturated fatty acids (PUFAs) leading to reactive aldehydes that are mutagenic and genotoxic (86). In a mouse model for myelodysplastic syndrome using *NUP98*-*HOXD13* (NHD13) transgenic mice, increased levels of ROS were detected in bone marrow nucleated cells accompanied by increased DNA double strand breaks supporting a connection between ROS and malignant transformation (87). In this line, a 5-year prospective registry study including 599 MDS patients revealed a deceased rate of progression to AML in patients treated with iron chelators (60). On the contrary, an earlier meta-analysis of Zeidan et al. did not confirm differences in the progression of MDS to AML with or without administration of iron chelators (88). Thereby, analyses might differ due to different MDS subgroups, observation periods and a potential selection bias for patients with longer predicted survival receiving iron chelation. Deducing from these results, leukemic transformation as a result of iron overload is a valid hypothesis but data are still ambiguous and more prospective trials are required. Possibly, disease related risk factors in MDS may overcome the influence of iron overload on progression to AML. The fact that mutations in the hereditary hemochromatosis protein (HFE) have not been found to increase the risk of AML (89, 90) may also indicate that *de novo* AML development is not induced by systemic iron overload.

ROS is known to highly influence hematopoiesis including hematopoietic stem cell state and function (91, 92). ROS is also involved in the regulation of various intracellular processes and signaling pathways (e.g. NF-κB, MAPK, PI3K-Akt, ubiquitination) as it is able to interact directly with proteins, ions and other molecules (93). Therefore, ROS might also influence stemness and proliferation of MDS and AML cells. Many molecular lesions related to MDS and AML development including mutations in *FLT3*, *NRAS/KRAS* and *IDH1/2* affect intracellular ROS production, thus potentially promoting ROS-mediated oncogenic signaling (94). Therefore, iron might impact intracellular signaling and cell fate decisions also by its influence on intracellular ROS signaling. Indeed, iron and associated proteins are involved in some of these signaling pathways as described in section *Potential Roles of Iron-Related Intracellular Proteins in AML and MDS*. However, studies further investigating this theory are needed.

In the extreme, iron overload with subsequent overwhelming accumulation of ROS can lead to ferroptosis, a non-apoptotic form of programmed cell death dependent on iron that differs from other regulated cell death mechanisms as apoptosis. First labeled by Dixon in 2012, ferroptosis is the consequence of a reduced antioxidant defense leading to uncontrolled lipid peroxidation and subsequent oxidative cell death (95). Depending on the activation of ROS-connected signaling pathways, cells are at a different risk for ferroptosis. Treatment of NRAS-Q61L mutated AML cells with the ferroptosis-inducing molecule erastin resulted in enhanced ROS levels and cytosolic translocation of HMGB1 leading to cell death, whereas this effect was not seen in unmutated cell lines (96). Importantly, the effect was iron-dependent and *HMGB1* knock-down lead to lower expression of TFRC.

Leukemic cells seem especially exposed to iron overload with the risk of undergoing ferroptosis. This indicates that they may have gained some ferroptosis evasion strategies. Indeed, Hole et al. could show that higher levels of NOX-derived ROS (ROS) in AML blasts were tolerated by evading oxidative stress response through suppression of p38MAPK signaling (97). Additionally, glutathione peroxidases, which can protect cells from oxidative damage by reducing lipid hydroperoxides and free hydrogen peroxide are overexpressed in AML patient samples and associated with an adverse OS (98). Moreover, Yusuf et al. show a dependency of murine and human AML cells on ALDH3A2, which can detoxify fatty aldehydes and thereby prevent oxidative damage due to lipid peroxidation (99). In mouse models, reduction of Aldh3a2 induced ferroptosis in leukemic cells and was synergistically lethal combined with the inhibitor of glutathione peroxidase 4 (GPX4) RSL3, whereas it was dispensable for normal hematopoiesis. In this line, the transcription factor NFE2L2 also seems to strengthen the oxidative stress defense in leukemic cells by regulating the expression of many antioxidative proteins especially in case of additional chemotherapeutic treatment (100, 101). Parallelly, NFE2L2 also regulates the expression of iron-related proteins as FTH1, FTL and HMXO1 again supporting a close connection between ROS and iron homeostasis. All these findings support the hypothesis that AML cells might benefit from the toleration of higher iron and ROS levels. To which extent iron is involved in this pathomechanism and if this is also the case for MDS cells has to be further elucidated.

## Iron and the Microenvironment

Hematopoietic and leukemic blasts reside and proliferate in bone marrow niches interacting with their microenvironment. The microenvironment including mesenchymal cells, endothelial cells, sympathetic neurons, other hematopoietic and immune cells and the extracellular matrix is considered to be a key regulator of MDS and AML pathogenesis and recurrence (25, 102). Leukemic cells seem to adopt the bone marrow microenvironment according to their needs and suppress normal hematopoiesis *via* secretion of cytokines, microRNAs and exosomes.

Excess iron in AML and MDS patients is deposited in various organs including the bone marrow thereby altering the composition of the hematopoietic niche and potentially leading to hematopoietic niche defects. A murine iron overload model revealed elevated ROS levels and increased bone resorption leading to changes in the bone microarchitecture with trabecular and cortical thinning of the bone (103). This loss of bone substance seems related to changes in the bone marrow mesenchymal stem cells (BM-MSCs). Several alterations in the bone marrow stroma cell number and composition have been reported which concur in the fact that iron overload reduces the differentiation into osteoblasts relative to other cell subtypes and reduces matrix calcification (103–105). Cheng et al. could also demonstrate a ROS-mediated cell death of mesenchymal cells due to iron overload mediated by the AMPK/MFF/DNM1L pathway triggering mitochondrial fragmentation and reducing ATP production (106). The alterations of the mesenchymal cell compartment were also shown to influence their supporting function for hematopoiesis. Thereby, the expression of several adhesion molecules and cytokine secretion was altered in bone marrow stroma cells under overload conditions impairing their capacity to support hematopoietic cells growth (24, 105, 107). This might also be important for transplant engraftment during HSCT, as transplantation from normal donor mice to mice with iron overload resulted in a delayed hematopoietic reconstitution (107). Therefore, the effects of iron overload on bone marrow structure and mesenchymal cells might attribute to the defective hematopoiesis found in MDS and AML patients.

Macrophages in the bone marrow of MDS patients were shown to have higher FTH expression (108). Additionally, expression of HMOX1 in macrophages was associated with an adverse patient outcome. In the microenvironment of solid tumors, tumor-associated macrophages (TAMs) are thought to contribute to tumor progression *via* delivery of iron to the tumor cells by an iron-release macrophage phenotype (109). However, it has not been investigated if there might be a similar role of leukemia-associated macrophages.

Normal cells of the hematopoietic and especially erythropoietic system are also highly affected by changes in the iron homeostasis as already described in section *Iron Homeostasis and its Role for Normal Hematopoiesis*. Morbidity and mortality in iron overloaded MDS and AML patients might therefore largely be explained by the toxicity of iron to these cells. In a murine iron overload model using RUNX1S291fs-induced MDS mice, the survival of iron overloaded MDS mice decreased as a result of an impaired frequency and colony-forming capacity of normal hematopoietic stem cells (110).

The iron household in the bone marrow might also affect endothelial cells and the vascular architecture. Cellular iron deficiency increases VEGF-induced angiogenesis (111, 112). Moreover, it has been shown that ferritin promotes the assembly of endothelial cells by antagonizing the antiangiogenic effects of cleaved high molecular weight kininogen (113).

Beside the bone marrow, an altered iron metabolism can also impact other organs. Iron overload due to multiple transfusions has been demonstrated to be toxic to various organs as liver, heart, pancreas, thyroid and pituitary gland leading to an increased morbidity and mortality (114). The influence of organ iron overload on patient outcome in MDS and AML patients is not fully determined yet. As described in section *Elevated Iron Levels in MDS and AML Patients*, data on a potential correlation of liver iron overload with NRM are ambiguous.

## Iron, Inflammation, and Infection

Iron and proteins related with iron overload are closely connected to local or systemic inflammation and might also influence the occurrence of infections by effects on the immune system and various pathogens.

Patients with AML and MDS are often immunocompromised due to a suppression of normal hematopoiesis by the disease and bone marrow toxicity of applied chemotherapies. Additionally, the patients frequently undergo multiple medical interventions including placements of catheters, which further increase the risk of inflammation and infection. Patients receiving an allogeneic HSCT have also a risk of inflammation due to a Graft-versus-Host Disease (GvHD) and need immunosuppressive therapy.

Inflammatory stimuli lead to an upregulation of hepcidin and other acute-phase proteins as ferritin and caeruloplasmin as well as a down-regulation of negative acute-phase-proteins as transferrin. The resulting downregulation of available plasma iron may withhold iron from pathogenic microorganisms and protect healthy tissues from ROS damage at the site of infection. Many microorganisms require iron for electron transport, glycolysis, genome synthesis and defense, making it an essential nutrient. Excess iron has shown to stimulate the growth of many gram-positive and gram-negative bacteria, fungi and single-celled eukaryotes as well as the replication of viruses (115, 116). Correspondingly, patients with hemochromatosis or hemoglobinopathies are at increased risk for infectious diseases due to iron overload (117, 118).

In patients undergoing allogeneic HSCT, high pre-transplant ferritin levels have been associated with an increased risk for invasive fungal pneumonia (119, 120) and hepatosplenic candidiasis (121). Patients suffering from mucormycosis in allogeneic HSCT recipients were found to have a severe iron overload compared with a matched control population (122). Moreover, early bacterial infections in allogeneic HSCT recipients were increased in patients with elevated pre-transplant hepcidin levels (123). A large metanalysis demonstrated a higher incidence of blood stream infections, a lower incidence of chronic GvHD and no effect concerning acute GvHD to be associated with high-pretransplant ferritin levels (52). Additionally, Pullarkat et al. reported in a prospective study, that iron overload measured by pre-transplant ferritin was a risk factor for mortality and blood stream infections but also for acute GvHD (124). Thereby, all these studies point towards a prognostic impact of iron overload markers as ferritin and hepcidin for fungal and bacterial infections as well as for the occurrence of GvHD. Although an elevation of these markers was measured before the onset of the diseases, a bias for patients that were already initially prone to inflammation and infection cannot be excluded.

The function of cells belonging to the immune system may also be influenced by iron homeostasis. In MDS patients with iron overload measured by elevated ferritin and transferrin saturation, Chen et al. found a lower percentage of CD3+ T-cells and disrupted T-cell subsets accompanied by higher ROS-levels in these cells (125). Using a murine iron overload model, Chen et al. showed that iron overload could reduce peripheral T-cells, decrease Th1/Th2 as well as Tc1/Tc2 ratio and increase CD4/CD8 ratio as well as the fraction of regulatory T-cells by inducing ROS-mediated oxidative stress and apoptosis of T-lymphocytes. The impact of these alterations on the anti-leukemic defense, inflammation and infection as well as patient outcome is yet unclear.

## Therapies Aiming at Iron Metabolism as a Possible Target in MDS and AML

Features of iron overload, a differential iron metabolism and changes in proteins associated with iron have been found in MDS and AML patients. Markers of iron overload correlate with a worse prognosis in both patient cohorts. There is a rational for potential pathomechanisms explaining detrimental effects on the patient outcome by consequences of the altered iron metabolism. However, markers of iron overload are in many ways subject to the chicken-and-egg problem making it impossible to discriminate between cause and consequence. Therefore, interventional studies might cast light on the causative impact of the altered iron metabolism.

**Figure 3:**
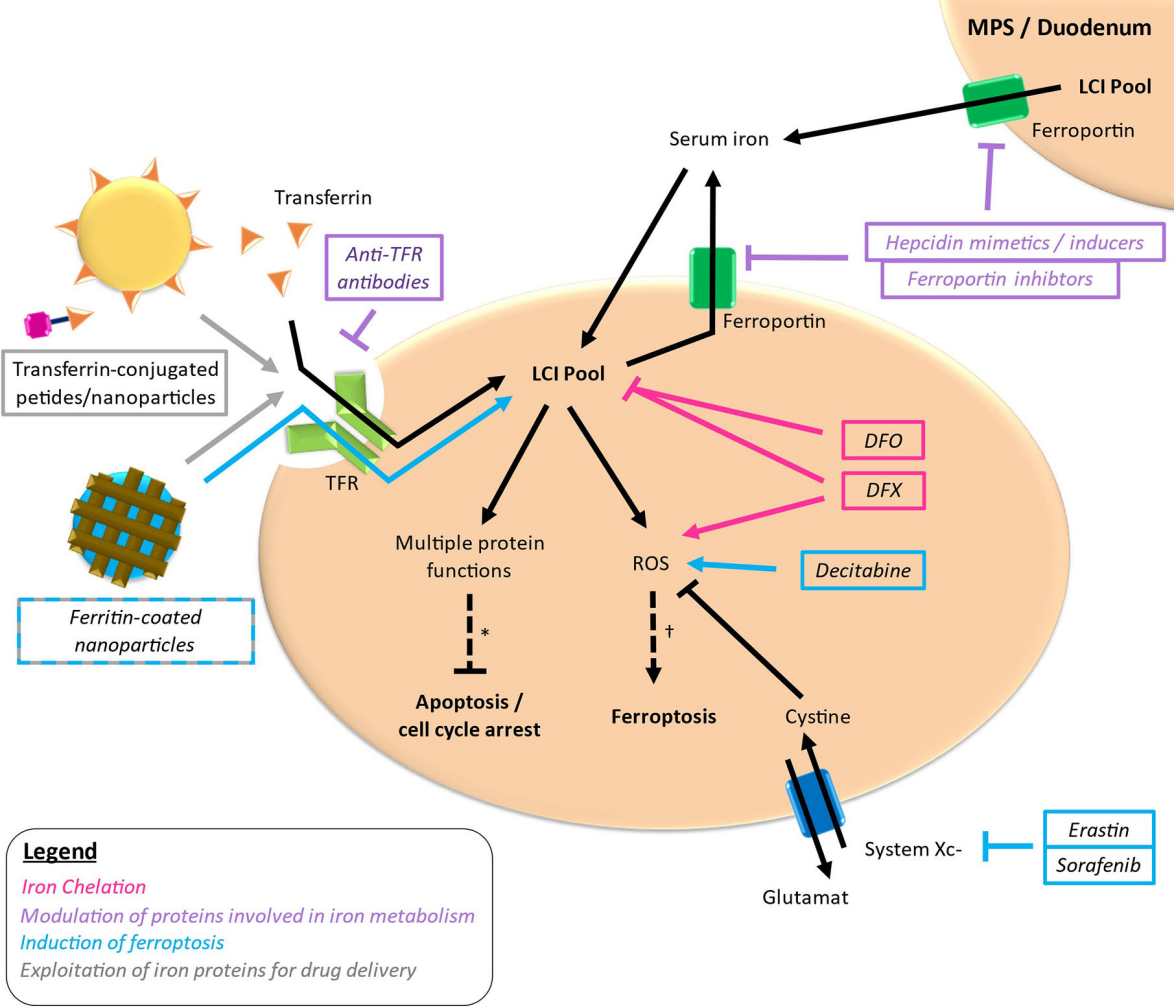
Targets of different drugs interfering with iron metabolism. Simplified outline with colored arrows indicating the respective way of action. *loss of important protein functions can induce apoptosis and cell cycle arrest. ^†^excessive ROS production leading to lipid peroxidation can lead to ferroptosis. LCI, labile cellular iron; TFR, transferrin receptor.

Iron-targeting strategies are based on the differential iron metabolism in case of MDS and AML compared to normal circumstances constituting a potential vulnerability in these diseases. Therapeutic strategies aiming at iron metabolism as a possible target in MDS and AML can be roughly distributed in four approaches: reduction of iron required for cellular functions *via* iron chelation, modulation of proteins involved in iron metabolism, induction of ferroptosis und exploitation of iron proteins for the delivery of antileukemic drugs (**Figure 3**). Thereby, most studies have been conducted using iron chelators, whereas the other approaches are in the early stages of development.

### Iron Chelation

Iron overload, whether or not caused by an impaired underlying, dysregulated mechanisms or by multiple red blood cell transfusions, has been demonstrated to influence many intracellular and systemic processes. The reduction of iron overload thus seems like an obvious therapeutic strategy to correct prognostically unfavorable effects.

Chelators can bind metal ions and afterwards be excreted as water-soluble complexes. By reducing NTBI, LPI and LCI pools, iron chelators may influence enzyme functions depending on iron, and influence ROS homeostasis. Therefore, iron chelation therapy (ICT) offers a rational therapeutic option in the treatment of patients with iron overload aiming at an induction of an antileukemic effect and a reduction of secondary organ dysfunctions and infections. So far, there are three iron chelators approved by the European Commission/EMA for the treatment of patients with iron overload: Deferoxamine (DFO) administered parenterally and the orally available deferiprone (DFP) and deferasirox (DFX). Whereas DFP is approved only for patients with thalassemia major, DFO and DFX have broader indications including iron overload in MDS and AML patients.

Iron chelators seem to act by various mechanisms. Deferoxamine (DFO) was shown to negatively affect DNA synthesis and reduce cell growth in the leukemic cell line K562 by impairing the activity of ribonucleotide reductase (126). Ribonucleotide reductase catalyzes the formation of deoxyribonucleotides and needs iron as a cofactor to build a tyrosyl radical crucial for its function. DFO was shown to inhibit the enzyme activity by depletion of the LCI pools necessary to regenerate the active enzyme (127). Moreover, iron chelators can affect ROS homeostasis in two opposite directions leading to either ROS depletion or ROS promotion (128). The ROS depleting effect is suggested to depend on diminished free labile iron levels (129), whereas the ROS promoting effect may be facilitated by an iron-mediated free radical generation through the iron-chelator-complex (130, 131) or by a potentially iron-unrelated induction of ROS signaling (132). The effect of ICT on ROS seems thereby to depend on the binding-characteristics of the chelator, the time of treatment and the used concentration (133). Both mechanisms seem to play a role in ICT activity. The ROS-promoting activity has been suggested to participate in the effect of DFX in AML cells (128, 133). On the contrary, oxidative stress was reduced under long-term DFX treatment in MDS patients with iron overload (134, 135). ICT is also reported to enhance the effect of other antiproliferative drugs. *In vitro* and *in vivo* studies showed an increased antileukemic effect for the combination of DFO and cytarabine (136), DFX and decitabine (137) as well as DFO and doxorubicin (138). A potential mode of action for the combination of DFX with doxorubicin might be an increase of the intracellular calcium resulting in an improved sensitivity to chemotherapy in leukemia cell lines (138). Moreover, ICT has been found to modulate different signaling pathways including a repression of mTOR and NF-kB signaling pathways, which might also explain a potential synergistic effect with other drugs (139, 140). Iron chelators were also shown to act synergistically with differentiating agents in the treatment of AML (133). Thereby, iron chelation led to ROS production, activation of MAPK pathways and also induced expression and phosphorylation of the vitamin D3 receptor (VDR) leading to blast differentiation *in vitro*, *in vivo* and also in one patient with secondary AML treated with DFX and vitamin D3 after relapse of the disease (133). Deducing from these results, the mechanism of ICT action might not solely be the iron-deprivation but rather also a modulation of ROS homeostasis and intracellular signaling. A relation of the latter effects with the iron-modulating activity seems likely, but iron-independent effects of the ICT cannot be excluded. The diverse effects might not only depend on the way of chelator administration but also on the status of the treated cells.

Clinically, there is some evidence from *post-hoc* analyses in cohorts of low/intermediate-1 risk MDS patients with iron overload that iron chelators as DFX may improve hematological parameters after administration over at least one year in a small proportion of the patients (141–145). An increase of hemoglobin, platelets and/or neutrophils was observed in 11%–22% of the patients with a few multilineage improvements and a few transfusion independencies. Thereby, the data of List et al. suggest a possible correlation between the amount of ferritin reduction by iron chelators and hematological response (143). In a retrospective analysis of 182 patients with MDS with various subtypes, the multivariate analysis revealed a significant benefit in OS for patients receiving ICT with 140.9 months vs 36.3 months (p=0.0008) in case of refractory anemia (RA or, according to the present WHO classification: MDS-RS), 133.4 months vs 73.3 months (p=0.02) in case of refractory anemia with ring sideroblasts (RARS/RARS-t, corresponding to MDS-RS according to the present WHO classification) and no difference for refractory cytopenia with multilineage dysplasia (RCMD/RCMD-RS, corresponding to MDS-MLD according to the present WHO classification) (146). The latter indicates that not all subtypes of MDS may benefit from ICT. It should also be noted that ICT seems to have the largest effects in subtypes which were suspected to suffer more from primary iron overload, MDS-RS and MDS-RA but not MDS-MLD, as marked by a reduced hepcidin/ferritin ratio described in section *Elevated Iron Levels in MDS and AML Patients*.

A recent systematic review and meta-analysis by Zeidan et al. included nine studies (4 prospective and 5 retrospective) with a total of 2450 patients with particularly low-risk MDS of whom 38.4% received ICT (88). Patients with ICT had a lower mortality and longer OS compared to no ICT with a pooled estimate of the ratio median OS of 2.1 years, suggesting that iron chelation therapy might double the OS in MDS. Additionally, there were some hints at a correlation between dose intensity of ICT and OS. Two of the reported studies compared patients with high adequate ICT to no ICT showing a highly significant survival advantage for patients with a higher adequate dose, but comparing any degree of ICT with no ICT, the OS benefit was less pronounced (88, 147, 148). In the study by Rose et al., adequate ICT was associated with median OS of 124 months compared to 85 months for ICT (p < 0.001) (147). Similar results were described by Delforge regarding OS with adequate ICT and no adequate ICT (p = 0.001) but not between weak ICT and no ICT (148). Hereby, adequate chelation was defined for DFO subcutaneously (40 mg/kg/day in slow infusion over 8–12 h for at least 3 days per week), DFX (20–30 mg/kg/day p.o.) or DFP (30–75 mg/kg/day p.o.); weak chelation treatment was considered to be less than 3 g per week of DFO. The question whether there are any differences regarding the efficacy between the iron chelators cannot be answered finally due to a lack of randomized trials. However, the compliance of DFX might be better than that of DFO or DFP due to the oral mode of administration and the less frequent occurrence of side effects resulting in a continued application and more remarkable reduction of iron overload (149–153). Gastrointestinal adverse events and neutropenia were more frequently observed in DFP than in DFO (149, 150).

Randomized trials in MDS looking for the clinical benefit using iron chelators in patients with excessive iron overload are highly needed. Recently, Angelucci et al. published data from the randomized clinical study TELESTO (154). Here, 225 patients with low- to intermediate-1 risk MDS were treated with DFX versus placebo in a 2:1 randomization. The event-free survival (EFS) was prolonged with 3.9 years in the DFX versus 3.0 years in the placebo arm (HR 0.64). Although the study is limited by an amendment from a planned phase 3 trial with 630 patients to a phase 2 trial with 225 patients and different follow-up times between the groups, the data again support a benefit of iron chelation on the clinical outcome.

There are some weak hints that iron chelation also has positive effects after allogeneic HSCT on hematological reconstitution, but the number of patients reported is limited. So, in a rather small cohort of eight patients with incomplete hematological reconstitution after allogenic HSCT, treatment with DFX led to hematological improvements with a subsequent loss of transfusion dependency in all patients within a maximum of 30 days (155). Moreover, Cho et al. propose an enhanced graft-versus-leukemia (GvL) effect leading to a lower incidence of relapse, an improvement of DFS and OS, while the incidence of chronic GvHD by DFX treatment post-transplant increases (156). The data, however, are limited due to their retrospective analysis.

Besides the iron chelators mentioned above, there are also new iron chelators and other substances with iron-chelating properties under investigation. In a phase 2 study, triapine, forming a potentially redox active iron complex and known to inhibit the M2 subunit of the ribonucleotide reductase, showed clinical activity when administered sequentially with fludarabine in patients with accelerated myeloproliferative diseases and secondary AML (157, 158). Ciclopirox olamine, an antimycotic agent with iron chelation activity, showed a hematologic improvement in 2 out of 23 patients with relapsed or refractory hematologic diseases in a phase 1 study (159). Moreover, eltrombopag, a thrombopoietin receptor agonist approved for the treatment of idiopathic thrombocytopenic purpura and aplastic anemia, has also shown to be an efficient iron chelator, mobilizing iron and reducing ROS working synergistically with other iron chelators *in vitro* (160). In a mechanistic study on HSCs, eltrombopag stimulated hematopoiesis at the stem cell level through iron chelation-mediated reprogramming (161). Randomized placebo-controlled phase 1/2 data revealed a reduction of clinically relevant thrombocytopenic events upon eltrombopag treatment in MDS and AML patients (162, 163). On the contrary, a subsequent randomized phase 2 trial investigated the receipt of eltrombopag during standard induction therapy in AML patients and found no clinical benefit of eltrombopag but rather a tendency for increased severe adverse events (164).

The clinical data demonstrate activity of ICT in the treatment of low/intermediate-1 risk MDS patients with iron overload suggesting a potency of ICT as an additional treatment option. The other way around, it can be deduced that iron overload in these patients might be accountable for a worse patient outcome. Thereby, ICT seems to specifically improve the hematopoietic response. There is only limited data on the effect of ICT on leukemic cells themselves and on the role of ICT in AML. Deducing from some preclinical studies, ICT might here influence intracellular signaling and ROS homeostasis specifically in combination with other drugs.

### Modulation of Proteins Involved in Iron Metabolism

Many different proteins are involved in iron metabolism and have demonstrated differential expression in MDS and AML cells as described in section *Potential Roles of Iron-Related Intracellular Proteins in AML* and *MDS*. Targeting these proteins therefore represents another potential treatment approach.

Considering that malignant cells need iron for proliferation and that TFR was demonstrated to be expressed on the surface of AML cells, it was tested if inhibition of the TFR may lead to an antiproliferative effect due to a decreased iron import. Indeed, various TFR antibodies showed inhibition of DNA synthesis and a subsequent growth inhibition of AML cells *in vitro* and a reduction of tumor growth in mouse models (126, 165–168). The effect of different TFR antibodies was even enhanced when used in combination (169). However, as TFR is also expressed on normal cells of the hematopoietic system and TFR antibodies have shown to impair growth of normal hematopoietic cells as well (165), bone marrow toxicity is thought to be an important side effect of the treatment. Despite this fact, administration of the TFR antibody 42/6 in patients with refractory cancer including lymphoma patients was well tolerated in a phase 1 trial (170). Clinical data for the treatment of MDS and AML patients are missing.

Hepcidin as regulator of systemic iron provides another reasonable antileukemic target with the aim to reduce overall iron load and subsequent toxic effects on organs as heart, liver and bone marrow. Hepcidin as a potential target of iron-homeostasis has been investigated in iron overload situations but without specific data for MDS and AML. Synthetic hepcidin mimetics such as PTG-300 or LJPC-401 have been reported to reduce serum iron levels and to be well-tolerated in phase 1 trials in healthy subjects and patients with iron overload, although the clinical relevance has still to be determined in ongoing studies (171, 172). Various other hepcidin targeting agents, for instance humanized monoclonal antibodies (LY2787106; 12B9m), the anticalin (PRS-080), and Lexaptepid Pegol (NOX-H94) have been tested in preclinical models or early in-human trials as reviewed by Crielaard et al., but failed major efficacy so that further development was stopped (173). Matripase-2 (MT2A), a transmembrane serine protease predominantly expressed in hepatocytes suppresses the expression of hepatic hepcidin by cleaving the membrane hemojuvelin into an inactive form (174). Antisense DNA (IONIS-TMPRSS6-LRx) or liposomal siRNA (ALN-TMP) as well as some protease inhibitors have demonstrated specific MT-2 inhibiting activity with the potential to reduce secondary anemia in patients with iron overload in preclinical models (173–176). Targeting the hepcidin-ferroportin pathway by inhibiting the bone morphogenic protein BMP6, which stimulates hepcidin expression in the liver or the iron exporter ferroportin *via* the monoclonal antibodies, LY3113593 and LY2928057, has not been further investigated beyond a phase 1 study (177). Therefore, data on the role of the hepcidin-ferroportin axis as a potential therapeutic target were mostly negative, further studies of MT-2 inhibitors have to be awaited.

### Induction of Ferroptosis

In contrast to influencing the course of the disease in MDS and AML by reducing iron overload, enhancing iron overload to induce ferroptosis represents an opposing but alterative mechanism. There are various agents acting as inhibitors or inducers of ferroptosis: Iron chelators, lipophilic antioxidants, inhibitors of lipid peroxidation and depletion of PUFAs inhibit ferroptosis, whereas ferroptosis is induced by the accumulation of iron or PUFA-phospholipids and by the depletion of endogenous inhibitors such as GSH, NADPH, GPX4 or vitamin E (178).

Erastin is a ferroptosis inducer acting on multiple levels. It inhibits the cysteine/glutamate antiporter system Xc-, thereby revoking cysteine import and thus reducing glutathione synthesis. It activates TP53, which can also inhibit system Xc, and it induces the opening of voltage-dependent anion channels (VDACs), thereby inducing mitochondrial dysfunction (179). The activation of ferroptosis by erastin promotes chaperone-mediated autophagy and the degradation of glutathione peroxidase 4 (GPX4) (180). In AML cell lines, erastin has shown a dose-dependent mixed-type of cell death, including ferroptosis, and enhanced the antileukemic effect of cytarabine and doxorubicin (156). Besides, the tyrosine kinase inhibitor sorafenib, which is approved for the treatment of liver renal and thyroid carcinoma and also showed efficacy in AML patients with FLT3-ITD (181, 182), also inhibits the system Xc^-^ (183).

Other ferroptosis inducers have shown antileukemic activity in AML cells as well: Dihydroartemisinin (DHA) was shown to induce ferroptosis of AML cells by leading to accelerated degradation of ferritin and increasing LPI (184). Besides, the frequently used antileukemic drug decitabine has recently suggested to induce ferroptosis (185). Treatment of MDS/AML cell lines with decitabine increased ROS levels by reducing GSH and GPX4 activity. Ferroptosis inducers enhanced the effect of decitabine, whereas ferroptosis inhibitors abrogated the effect. As iron chelators also potentiated the effect of decitabine, this is another hint that treatment effects may be mediated by ROS and might also be influenced by the intracellular iron household.

The data suggest a potential use of ferroptosis inducers in the treatment of AML, although clinical data are missing. There are not enough data to estimate the role of ferroptosis induction in MDS.

### Exploitation of Iron Proteins for Targeted Drug Delivery

Another attempt to specifically target malignant cells is to use the TFR as target protein for the delivery of another tumor-specific cargo. Covalent conjugates of the ferroptosis inducing agent artemisinin and a transferrin-receptor targeting peptide combined ferroptosis induction and targeted delivery and revealed antileukemic affectivity *in vitro* (186). Thereby, artemisinin could be co-internalized with receptor-bound transferrin and could use the iron deliberated by transferrin to generate cytotoxic ROS. Moreover, transferrin-conjugated nanoparticles have shown potential in the delivery of antileukemic drugs: Transferrin-conjugated lipid nanoparticles delivering an antisense oligonucleotide targeting *BCL2* mRNA induced caspase-dependent apoptosis in AML cell lines and suppressed tumor growth of human AML xenograft tumors in mice (187, 188). Transferrin-conjugated liposomal nanoparticles containing antagomiR-126 resulted in reduction of leukemic stems cells in an AML mouse model (189). Additionally, transferrin-conjugated nanoparticles delivering doxorubicin showed cytotoxicity in myeloid leukemia cells *in vitro* and *in vivo* (190, 191). Also, transferrin-conjugated polymeric nanoparticles delivering edelfosine and lipid-based nanoparticles delivering etoposide revealed antileukemic activity *in vitro* (192, 193).

Ferritin can also be used as a protein cage for the delivery of other molecules due to its tertiary structure (194). As FTH can be bound and uptaken by TFRC (20), this provides another way of directed targeting. Ferritin nanovesicles delivering cytochrome C induced apoptosis in a promyelocytic AML cell line (195). Delivery of cytarabine in form of Fe_3_O_4_@SiO_2_-cytarabine nanoparticles increased the cytotoxic effect of cytarabine alone about two orders of magnitude in cell lines (196). The combination of erastin and rapamycin, an inducer of autophagy, with ferritin as a nanodrug showed increased inhibition of tumor growth compared to the drugs administered separately (197). Besides, use of iron saturated ferritin as a component of nanoparticles may also contribute to ferroptosis induction. The intravenous iron preparation ferumoxytol has also shown to increase ROS and thereby induce ferroptosis in patient derived xenografts from primary AML samples with low ferroportin (198). Furthermore, nanoparticles using Fenton reactions to improve ferroptosis are under investigation (199).

Taken together, a couple of possible therapeutic agents have been developed that hijack iron proteins for target delivery. Their effectivity has been demonstrated *in vitro* and *in vivo*. Clinical studies have to further evaluate their use in patients.

## Perspectives

In this review, we demonstrated the clinical significance of iron homeostasis in MDS and AML patients. Iron metabolism has been shown to impact multiple intracellular functions, the production of ROS, the microenvironment as well as the susceptibility to infections. Markers of iron overload were demonstrated to have prognostic relevance although the impact of an altered iron metabolism on patient outcome in MDS and AML is still under debate as markers of iron overload are highly influenced by inflammatory signals and complicate the detection of causative associations. Supporting a partially causative connection between iron metabolism and patient outcome, therapeutics addressing the iron balance as ICT were found to improve the outcome especially in low/intermediate-1 risk MDS patients. As recurrent red blood cell transfusions constitute the major source of secondary iron overload in MDS and AML patients, a more restrictive application should be considered. Moreover, various agents targeting proteins involved in iron homeostasis or inducing ferroptosis are investigated preclinically or are in early clinical development. With a more detailed understanding of the pathophysiology of MDS and AML in the context of iron, future development of new iron-targeting strategies may lead to better patient outcomes. Therefore, basic research further investigating the processes involved in iron homeostasis linked with redox balance and leukemia is inevitable. Moreover, clinical studies analyzing reliable markers for pathophysiological active iron overload and prospective studies exploring function of iron-homeostasis targeting drugs are essential. Especially the combination of iron-homeostasis targeting drugs with other antileukemic agents constitutes a promising approach due to potential synergistic effects and should therefore be further elucidated.

## Author Contributions

Conceptualization was done by SW, AP, and HS. Investigation and writing of the draft was done by SW and AP. Review and editing of the draft was done by NK, FS, and HS. Supervision was done by HS. All authors contributed to the article and approved the submitted version.

## Funding

This work was supported by the German Cancer Consortium (DKTK) (SW, HS), the Alfred and Angeliga Gutermuth-Stiftung (SW), the Deutsche Forschungsgemeinschaft (DFG, German Research Foundation)—SFB1177 TP E07 (HS) and SFB815 TP A10 (HS, NK) and by the LOEWE Center Frankfurt Cancer Institute (FCI) funded by the Hessen State Ministry for Higher Education, Research and the Arts [III L 5-519/03/03.001-(0015)].

## Conflict of Interest

The authors declare that the research was conducted in the absence of any commercial or financial relationships that could be construed as a potential conflict of interest.

## Abbreviations

AML, acute myeloid leukemia; ALL, acute lymphoblastic leukemia; DFS, disease-free survival; GvHD, graft-versus-host disease; HFE, hereditary hemochromatosis protein; HSCT, hematopoietic stem cell transplantation; ICT, iron chelation therapy; IRE, iron-responsive elements; IRP, iron-responsive element binding protein; LCI, labile cellular iron; LPI, labile plasma iron; MDS, myelodysplastic syndrome; NRM, non-relapse mortality; NTBI, non-transferrin-bound iron; OS, overall survival; PFS, progressive-free survival; PUFA, polyunsaturated fatty acid; RFS, relapse-free survival; ROS, reactive oxygen species; TAM, tumor-associated macrophage; TCA, tricarboxylic acid cycle; TFR, transferrin receptor.
